# The Relationship Between Grip Strength and Cognitive Impairment: Evidence From NHANES 2011–2014

**DOI:** 10.1002/brb3.70381

**Published:** 2025-03-18

**Authors:** Wenyi Nie, Jingqing Hu

**Affiliations:** ^1^ Department of Traditional Chinese Medicine Changchun University of Chinese Medicine Changchun China; ^2^ Tianjin University of Traditional Chinese Medicine Tianjin China

**Keywords:** cognitive impairment, grip strength, muscle strength, NHANES, older adults

## Abstract

**Background:**

Prior research has consistently shown an association between muscle strength and various metabolic diseases. However, the relationship between muscle strength and cognitive impairment remains elusive.

**Methods:**

Using data from the 2011–2014 National Health and Nutrition Examination Survey (NHANES), we investigated the association between muscle strength and cognitive impairment. We used multivariate logistic regression, restricted cubic spline regression (RCS), and threshold effects to investigate the impact of muscle strength on cognitive function. In addition, we analyzed the correlation between muscle strength and cognitive impairment in subgroups of age, gender, race, education, smoking status, drinking status, hypertension, diabetes, and heart disease.

**Results:**

2124 participants were ultimately included for further analysis. Multivariate logistic regression, trend testing, and RCS analysis showed a non‐linear negative correlation between muscle strength and cognitive impairment. Threshold effect analysis suggests that when muscle strength reaches a certain value, this relationship undergoes significant changes. In the three cognitive function scoring tests, interaction was only observed in the racial subgroup.

**Conclusion:**

This study suggests a negative correlation between muscle strength and cognitive function, which may have a threshold effect. Further longitudinal studies are needed to elucidate its potential mechanisms.

## Graphical abstract

This diagram illustrates that exercise can enhance muscle strength, which in turn affects brain function, particularly cognitive function. In summary, the research findings suggest that greater muscle strength, as measured by grip strength, is associated with a reduced risk of cognitive impairment.

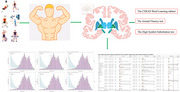

## 1 Introduction

With the continuous improvement of the average life expectancy of the global population and the increasingly severe aging problem, the rapid growth of the elderly population has brought unprecedented challenges to the current society and economy, especially the public health system (Sanchez‐Rodriguez et al. 2019, Frith and Loprinzi 2018). Mild cognitive impairment (MCI) is a transitional stage from healthy cognitive aging to dementia, characterized by decreased memory and cognitive decline (Boyle et al. 2006). Although there are currently no specific drugs that can slow down or cure MCI, adjusting lifestyle, including diet, physical exercise, psychological activity, and social interaction, may have a positive impact on preventing cognitive decline (Petersen 2016). Therefore, identifying and intervening in variable cognitive impairment risk factors is particularly important for developing prevention strategies to mitigate their negative impacts.

Grip strength, as a reliable, simple, fast, and economical muscle strength assessment indicator, is closely related to physical activity and may be associated with cognitive function (Peolsson et al. 2001). A study by the University of Sydney (Mavros et al. 2017) suggests that muscle strength training can improve brain function, especially beneficial for individuals aged 55 and above with MCI. A research team from the University of California, San Francisco (Duchowny et al. 2022), found that for every 5 kg decrease in grip strength, the risk of developing various cognitive disorders significantly increases. However, Ritchie et al.’s study did not observe a direct correlation between grip strength and cognitive function (Ritchie et al. 2016).

In summary, the current research conclusions on the relationship between grip strength and cognitive dysfunction are not unified. This study utilized a large dataset from the National Health and Nutrition Examination Survey (NHANES) from 2011 to 2014 and employed more comprehensive statistical methods to explore the potential association between grip strength and cognitive impairment in elderly Americans. This study is expected to deepen our understanding of muscle strength as a potential biomarker of cognitive health and provide a scientific basis for developing strategies to protect cognitive function in the elderly population.

## 2 Materials and Methods

### 2.1 Study Population

The data for this study was sourced from the NHANES, a nationwide health and nutrition survey conducted jointly by the National Institutes of Health and the Centers for Disease Control and Prevention. NHANES aims to comprehensively assess the health level and nutritional intake of the American population, providing a solid scientific basis for the formulation of public health policies and the implementation of health interventions. The research design of this survey project has been officially approved by the Institutional Review Board of the National Center for Health Statistics in the United States, and all individuals participating in the survey have signed informed consent forms; NHANES' database is open to the public and allows direct use for related data analysis (Mao et al. 2024, Zou et al. 2024). For more detailed information about NHANES, please visit the official website(https://www.cdc.gov/nchs/nhanes/about_nhanes.htm).

**FIGURE 1:**
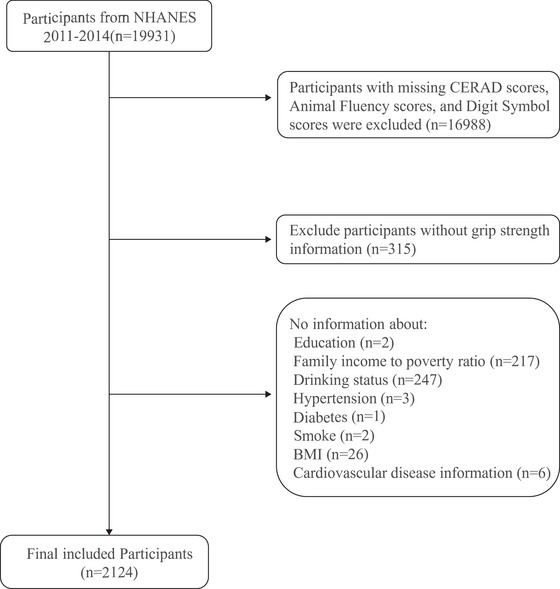
Flowchart of participants in this study.

For this study, 19,931 individuals were included initially. After applying strict exclusion criteria, 17807 participants were excluded, resulting in a final sample of 2124 individuals for analysis. The exclusion criteria included (1) people lacking cognitive testing data, (2) people with missing muscle strength data, and (3) a population with missing covariate data. The specific exclusion conditions are shown in Figure 1.

### 2.2 Cognitive Impairment Assessment

The cognitive abilities of the participants were assessed through a series of tests within the NHANES, encompassing the consortium to establish a registry for alzheimer's disease (CERAD) word learning and recall modules, the animal fluency test (AFT), and the digit symbol substitution test (DSST). Specifically, the CERAD evaluated participants' capacity to swiftly learn and recall new verbal information, the AFT assessed categorical verbal fluency, and the DSST measured processing speed and attention. Consistent with prior research (Wang et al. 2024, Liu et al. 2024, Dong et al. 2020, Li et al. 2024), despite the absence of definitive thresholds for the DSST, CERAD, and AFT to indicate cognitive decline, this study utilized the 25th percentile of the scores as a benchmark to identify cognitive dysfunction.

### 2.3 Grip Strength

Previous studies (Bohannon 2019, Yeung et al. 2018, Bohannon 2019) have validated grip strength as a reliable proxy for general muscle function and an important predictor of health outcomes. In this study, an established measure of muscle strength was recorded as the mean of multiple grip strength tests. Due to the skewed distribution of muscle strength data, we chose to logarithmize it to reduce data bias (Chamberlain et al. 2019, Zhou et al. 2016) and divide it into quartiles: < 1.67 kg, 1.67‐1.76 kg, and 1.77‐1.88 kg.

## 3 Statistical Analyses

All the statistical analyses adhered to the NHANES analysis guidelines. Following the NHANES methodology, WTMEC2YR was used as the weighting variable. Continuous variables were summarized using means and standard deviations (SDs), whereas categorical variables were expressed as counts and weighted percentages (Su et al. 2022). To compare the differences between groups, chi‐square tests and student's t tests were applied. Multiple logistic regression analyses were conducted to investigate the relationship between grip strength and cognitive impairment, with adjustments for potential confounders. Model 1 served as the unadjusted model, whereas model 2 was adjusted for age, sex, race/ethnicity, education, family income‒poverty ratio, and BMI (Body Mass Index). Model 3 was further adjusted for lifestyle factors, including drinking status, smoking status, hypertension, and diabetes, and model 4, the final model, included adjustments for cardiovascular disease and other health factors. Restricted cubic spline (RCS) analysis was employed to examine the potential nonlinear relationship between grip strength and cognitive impairment risk. Additionally, the subgroup and interaction analyses were conducted to explore how the relationship varied across different demographic and health‐related groups. Missing data were addressed via multiple imputations to maintain the sample size and mitigate potential bias (Carson et al. 2023). All analyses were conducted via IBM SPSS (International Business Machines Statistical Package for the Social Sciences) Statistics (version 24.0) and R software (version 4.3.0), with statistical significance set at p < 0.05 (Kong et al. 2024, Mao et al. 2024).

## 4 Result

### 4.1 Population Characteristics

Table 1 lists the characteristics of the study participants. A total of 2124 participants were included in this study, with an average age of 69.23 years. Of these participants, 49.58% were female and 50.42% were male. However, compared with the participants with grip strength Q1, the proportion of men increased with the gradual increase of grip strength, and there were significantly more men than women in the participants with grip strength Q4. Except for education, diabetes and heart disease, the differences of all variables included in this study were statistically significant in the four populations. Surprisingly, three different cognitive function test scores showed different performances in four populations. Specifically, the CERAD trial showed no significant difference among the four populations, but the other two trials were statistically significant.

**TABLE 1:** Characteristics of NHANES (2011–2014) participants with quartile log‐grip strength.

Variables	Total #(n = 2124)	Q1 (n = 529)	Q2 (n = 532)	Q3 (n = 532)	Q4 (n = 531)	*P*
Age(year)^a^, Mean ± SE	69.23 ± 6.72	72.16 ± 6.89	68.51 ± 6.46	69.25 ± 6.75	67.02 ± 5.66	< 0.001
Sex^b^, n(%)						< 0.001
Female	1053 (49.58)	487 (92.06)	408 (76.69)	152 (28.57)	6 (1.13)	
Male	1071 (50.42)	42 (7.94)	124 (23.31)	380 (71.43)	525 (98.87)	
Race/ethnicity^b^, n(%)						< 0.001
Mexican American	169 (7.96)	41 (7.75)	45 (8.46)	44 (8.27)	39 (7.34)	
Other hispanic	208 (9.79)	65 (12.29)	62 (11.65)	47 (8.83)	34 (6.40)	
Non‐hispanic white	1089 (51.27)	308 (58.22)	271 (50.94)	258 (48.50)	252 (47.46)	
Non‐hispanic black	472 (22.22)	68 (12.85)	99 (18.61)	134 (25.19)	171 (32.20)	
Other race	186 (8.76)	47 (8.88)	55 (10.34)	49 (9.21)	35 (6.59)	
Education^b^, n(%)						0.105
<9th grade	210 (9.89)	59 (11.15)	45 (8.46)	55 (10.34)	51 (9.60)	
9–11th grade	264 (12.43)	76 (14.37)	58 (10.90)	65 (12.22)	65 (12.24)	
High school diploma/GED	498 (23.45)	136 (25.71)	126 (23.68)	121 (22.74)	115 (21.66)	
Some college/AA degree	628 (29.57)	157 (29.68)	170 (31.95)	145 (27.26)	156 (29.38)	
≥ College graduate	524 (24.67)	101 (19.09)	133 (25.00)	146 (27.44)	144 (27.12)	
Family income to poverty ratio^a^, mean ± SE	2.68 ± 1.61	2.27 ± 1.52	2.75 ± 1.64	2.65 ± 1.58	3.05 ± 1.62	< 0.001
BMI^a^, mean ± SE	29.15 ± 6.36	28.58 ± 6.65	29.43 ± 6.92	28.99 ± 6.09	29.61 ± 5.68	0.038
Hypertension^b^, n(%)						0.003
No	822 (38.70)	169 (31.95)	214 (40.23)	221 (41.54)	218 (41.05)	
Yes	1302 (61.30)	360 (68.05)	318 (59.77)	311 (58.46)	313 (58.95)	
Diabetes^b^, n(%)						0.478
No	1546 (72.79)	375 (70.89)	383 (71.99)	404 (75.94)	384 (72.32)	
Yes	483 (22.74)	131 (24.76)	125 (23.50)	109 (20.49)	118 (22.22)	
Borderline	95 (4.47)	23 (4.35)	24 (4.51)	19 (3.57)	29 (5.46)	
Drinking status^b^, n(%)						< 0.001
No	338 (15.91)	153 (28.92)	91 (17.11)	61 (11.47)	33 (6.21)	
Yes	1786 (84.09)	376 (71.08)	441 (82.89)	471 (88.53)	498 (93.79)	
Smoking status^b^, n(%)						< 0.001
No	1848 (87.01)	483 (91.30)	469 (88.16)	457 (85.90)	439 (82.67)	
Yes	276 (12.99)	46 (8.70)	63 (11.84)	75 (14.10)	92 (17.33)	
Cardiovascular disease^b^, n(%)						0.316
No	1662 (78.25)	399 (75.43)	425 (79.89)	419 (78.76)	419 (78.91)	
Yes	462 (21.75)	130 (24.57)	107 (20.11)	113 (21.24)	112 (21.09)	
CERAD						
Score trial 1 recall^a^, mean ± SE	9.48 ± 216.86	4.64 ± 1.72	23.73 ± 433.30	4.77 ± 1.72	4.76 ± 1.56	0.382
Score trial 2 recall^a^, mean ± SE	16.19 ± 306.55	44.43 ± 613.83	7.08 ± 1.74	6.62 ± 1.78	6.77 ± 1.70	0.113
Score trial 3 recall^a^, mean ± SE	26.43 ± 433.29	64.19 ± 751.00	7.88 ± 1.72	26.23 ± 433.19	7.60 ± 1.66	0.112
Score delayed recall^a^, mean ± SE	24.85 ± 433.36	62.55 ± 751.12	25.19 ± 433.24	5.84 ± 2.30	6.01 ± 2.19	0.110
Animal fluency: score total^a^, mean ± SE	17.02 ± 5.56	15.94 ± 5.13	17.20 ± 5.52	17.07 ± 5.63	17.85 ± 5.80	< 0.001
Digit symbol: score^a^, mean ± SE	47.35 ± 17.01	44.04 ± 16.92	50.51 ± 18.08	46.31 ± 16.54	48.54 ± 15.78	< 0.001

Abbreviations: a: ANOVA, b: Chi‐square test, SE: standard error.

### 4.2 Relationship Between Grip Strength and Cognitive Impairment Risk

Table 2 summarizes the associations between log‐grip strength and cognitive impairment risk across different diagnostic methods. When analyzing log‐grip strength as a continuous variable, according to the unadjusted model, lower log‐grip strength was associated with a significantly greater risk of cognitive impairment. This association remained significant after controlling for confounders in model 4, which were adjusted for age, sex, race/ethnicity, education, income, BMI, lifestyle factors, and cardiovascular comorbidities (adjusted OR _CERAD_ = 0.13, 95% CI: 0.04–0.44; adjusted OR _Animal Fluency_ = 0.23, 95% CI: 0.07–0.75; adjusted OR _Digit Symbol_ = 0.02, 95% CI: 0.01–0.10). In CERAD and Digit Symbol tests, cognitive impairment gradually decreased with increasing grip strength when dividing grip strength into quartiles. However, this relationship does not seem to exist in animal fluency testing.

**TABLE 2:** Relationship between log‐grip strength and risk of cognitive impairment under different diagnostic methods.

Variables	Model1			Model2			Model3			Model4	
	OR (95%CI)	*P*		OR (95%CI)	*P*		OR (95%CI)	*P*		OR (95%CI)	*P*
CERAD											
Log‐grip strength	0.48 (0.24 ∼ 0.98)	0.044		0.12 (0.04 ∼ 0.39)	<.001		0.13 (0.04 ∼ 0.42)	<.001		0.13 (0.04 ∼ 0.44)	0.001
Log‐grip strength group											
< 1.67	1.00 (Reference)			1.00 (Reference)			1.00 (Reference)			1.00 (Reference)	
1.67‐1.76	0.60 (0.45 ∼ 0.81)	< 0.001		0.67 (0.47 ∼ 0.95)	0.023		0.68 (0.48 ∼ 0.97)	0.032		0.69 (0.49 ∼ 0.97)	0.034
1.77‐1.88	0.94 (0.71 ∼ 1.23)	0.635		0.60 (0.40 ∼ 0.90)	0.014		0.61 (0.41 ∼ 0.93)	0.020		0.62 (0.41 ∼ 0.94)	0.025
> 1.88	0.78 (0.59 ∼ 1.03)	0.079		0.52 (0.32 ∼ 0.83)	0.006		0.53 (0.33 ∼ 0.85)	0.008		0.54 (0.33 ∼ 0.86)	0.010
P for trend	0.374			0.007			0.010			0.012	
Animal fluency											
Log‐grip strength	0.24 (0.12 ∼ 0.50)	< 0.001		0.18 (0.06 ∼ 0.59)	0.005		0.22 (0.07 ∼ 0.74)	0.014		0.23 (0.07 ∼ 0.75)	0.015
Log‐grip strength group											
< 1.67	1.00 (Reference)			1.00 (Reference)			1.00 (Reference)			1.00 (Reference)	
1.67‐1.76	0.64 (0.48 ∼ 0.85)	0.002		0.78 (0.56 ∼ 1.08)	0.128		0.80 (0.58 ∼ 1.11)	0.182		0.80 (0.58 ∼ 1.11)	0.182
1.77‐1.88	0.72 (0.54 ∼ 0.95)	0.023		0.75 (0.50 ∼ 1.10)	0.140		0.79 (0.53 ∼ 1.18)	0.249		0.80 (0.54 ∼ 1.18)	0.258
> 1.88	0.66 (0.49 ∼ 0.88)	0.004		0.75 (0.47 ∼ 1.20)	0.228		0.80 (0.50 ∼ 1.29)	0.369		0.81 (0.50 ∼ 1.30)	0.381
P for trend	0.009			0.204			0.348			0.360	
Digit Symbol											
Log‐grip strength	0.23 (0.11 ∼ 0.47)	< 0.001		0.02 (0.00 ∼ 0.07)	< 0.001		0.02 (0.01 ∼ 0.09)	< 0.001		0.02 (0.01 ∼ 0.10)	< 0.001
Log‐grip strength group											
< 1.67	1.00 (Reference)			1.00 (Reference)			1.00 (Reference)			1.00 (Reference)	
1.67‐1.76	0.57 (0.43 ∼ 0.76)	< 0.001		0.54 (0.36 ∼ 0.80)	0.002		0.56 (0.38 ∼ 0.83)	0.004		0.56 (0.37 ∼ 0.83)	0.004
1.77‐1.88	0.77 (0.58 ∼ 1.01)	0.062		0.35 (0.22 ∼ 0.56)	< 0.001		0.38 (0.24 ∼ 0.61)	< 0.001		0.38 (0.24 ∼ 0.61)	< 0.001
> 1.88	0.55 (0.41 ∼ 0.73)	< 0.001		0.20 (0.12 ∼ 0.35)	< 0.001		0.22 (0.12 ∼ 0.38)	< 0.001		0.22 (0.12 ∼ 0.38)	< 0.001
P for trend	< 0.001			< 0.001			< 0.001			< 0.001	

Abbreviations: CI: Confidence Interval.

Model1: Crude.

Model2: Adjust: Sex, Age, Race/ethnicity, Education, Family income to poverty ratio, BMI.

Model3: Adjust: Sex, Age, Race/ethnicity, Education, Family income to poverty ratio, BMI, Drinking status, Smoking status, Hypertension, Diabetes.

Model4: Adjust: Sex, Age, Race/ethnicity, Education, Family income to poverty ratio, BMI, Drinking status, Smoking status, Hypertension, Diabetes, Cardiovascular disease.

### 4.3 Subgroup Analysis

**FIGURE 2:**
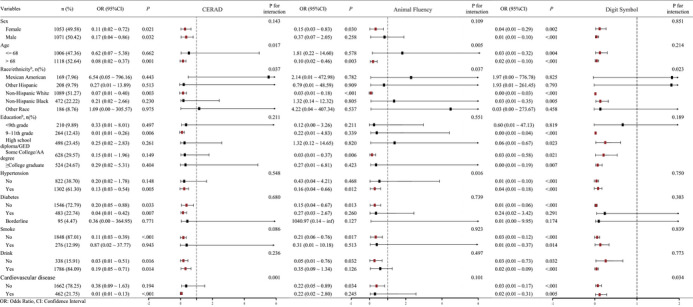
Subgroup analysis of grip strength and risk of cognitive impairment under different diagnostic methods.

The subgroup analysis results showed that (Figure 2) there was an interaction between age, race, and history of heart disease subgroups when using the CERAD test to assess cognitive impairment. There is an interaction between age, race, and history of hypertension subgroups when evaluating cognitive impairment using animal fluency testing. There is an interaction between race and history of heart disease subgroups when evaluating cognitive impairment using the digit symbol test.

### 4.4 Nonlinear Relationship and Threshold Effect

**FIGURE 3:**
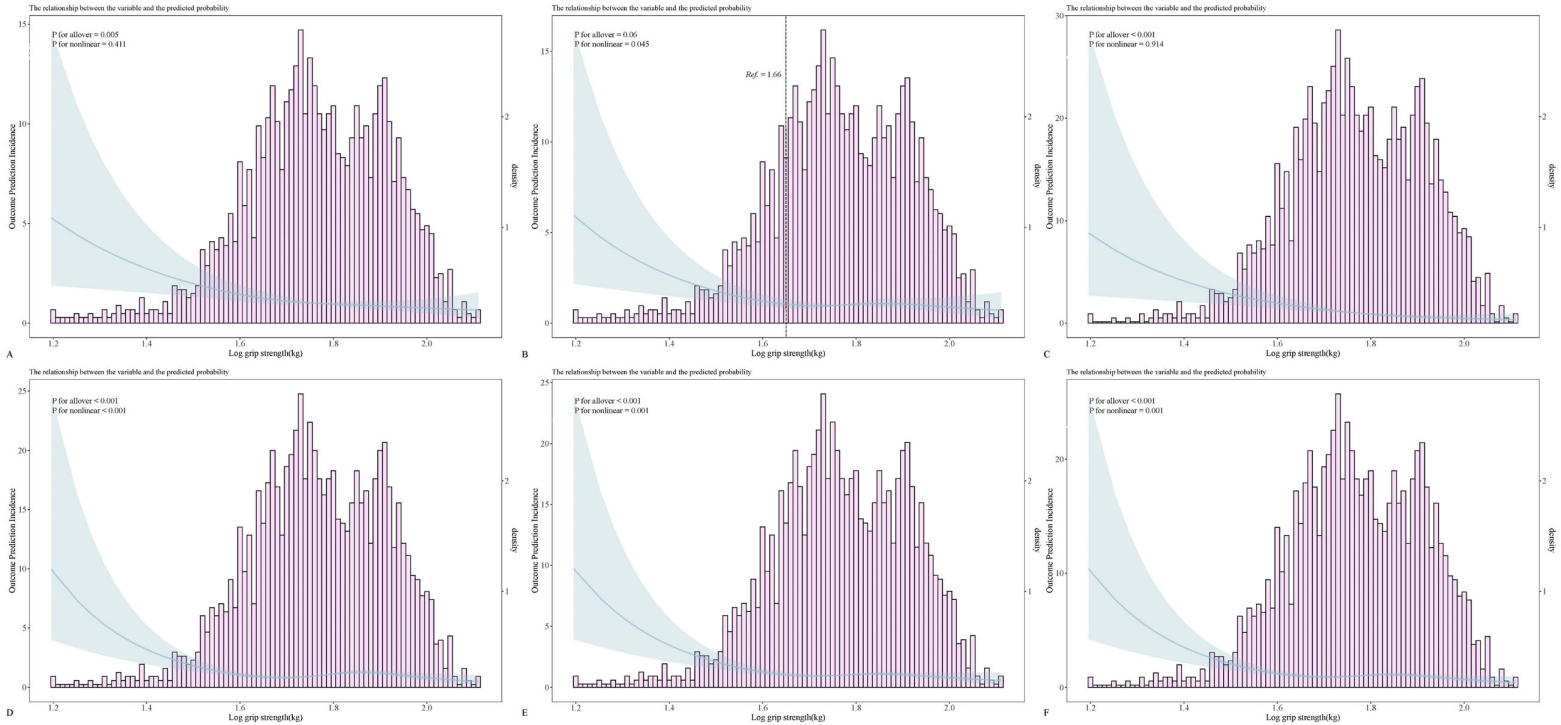
Non‐linear relationship between log‐grip strength and CERAD, AF, and DSST.

Due to the lack of a linear relationship between log‐grip strength and the risk of cognitive impairment in the multifactorial analysis of Animal Fluency testing, further evaluation is needed to determine if there is a non‐linear relationship between them. RCS analysis revealed a nonlinear relationship between log‐grip strength and cognitive impairment risk (Figure 3). A threshold effect was identified (Table 3), with an inflection point at 1.66 log‐grip strength (45.71 kg). Below this threshold, each unit increase in log‐grip strength was associated with a 2% reduction in cognitive impairment risk (adjusted OR = 0.02, 95% CI: 0.01–0.19). Beyond this threshold, there is no significant relationship between the increase in log‐grip strength and cognitive impairment. These statistical analysis results showed that in the American population, there is a negative correlation between the grip strength of the elderly and cognitive dysfunction. Increasing grip strength may potentially become one of the methods to improve cognitive function in the elderly.

**TABLE 3:** Threshold effect analysis of relationship between log‐grip strength and cognitive function scores diagnosed by Animal Fluency.

	OR (95%CI)	p‐value
One—line linear regression model	0.23 (0.07 ∼ 0.75)	0.015
Two—piecewise linear regression model		
Inflection point	1.66	
< 1.66	0.02 (0.01 ∼ 0.19)	0.001
> 1.66	1.00 (0.19 ∼ 5.27)	0.998
Log—likelihood ratio test		0.011

Sex, Age, Race/ethnicity, Education, Family income to poverty ratio, BMI, Drinking status, Smoking status, Hypertension, Diabetes, Cardiovascular disease.

## 5 Discussion

This study analyzed data from the NHANES 2011‐2014 cycle to investigate the association between grip strength (a representative of muscle strength) and the risk of cognitive impairment in elderly Americans. The research results show that regardless of the method used to evaluate cognitive function, grip strength is negatively correlated with cognitive dysfunction. RCS analysis further revealed a non‐linear relationship between grip strength and the risk of cognitive impairment. However, the RCS analysis of the animal fluency test may suggest a threshold effect in the relationship between grip strength and the risk of cognitive impairment. The existence of this relationship may be related to the fact that almost all the population in the Q4 log‐grip strength section of the study population is male. The result of this threshold is consistent with previous studies (Lopez‐Bueno et al. 2022, Lopez‐Bueno et al. 2023, Celis‐Morales et al. 2018).

These findings are consistent with previous studies that identified grip strength as an important health indicator associated with various health outcomes (Kim et al. 2019, Filardi et al. 2022, Van Hulle et al. 2021). Several studies (Sanchez‐Rodriguez et al. 2019) have reported a significant association between sarcopenia and cognitive impairment, while others have identified decreased muscle strength as an early marker of cognitive decline (Frith and Loprinzi 2018). In previous studies, there has also been literature describing the relationship between muscle strength and cognitive dysfunction (Chai et al. 2024, Chang and Zhao 2024, Peng et al. 2024). However, the study population is all Chinese. This study is one of the first to reveal a non‐linear relationship between grip strength and the risk of cognitive impairment, with significant threshold effects. This threshold effect means that beyond a certain point, an additional increase in grip strength may have a decreasing protective effect on cognitive health (Ludyga et al. 2021, Lim et al. 2022, Kim et al. 2022).

The subgroup analysis results showed that there was an interaction between racial subgroups in all three cognitive function testing experiments, indicating that there may be differences in the risk of cognitive dysfunction among different races. The explanation of this difference involves complex factors at multiple levels, including biology, sociology, culture, and environment. Some studies (Avila et al., 2021 Garcia et al. 2021, Tan et al. 2022, Xiao et al. 2023) suggest that racial differences in cognitive function are mainly determined by social health factors, and genetic lineage does not play a major role. Among them, the education level is particularly evident. Meanwhile, studies have also shown that differences in cognitive abilities between and within races are related to genetics (Duchowny et al. 2022, Liu et al. 2024, Zhao et al. 2021). In addition, some studies (Wang et al. 2023, Fotiadis et al. 2024) suggest that this difference is related to the differences in brain structure between different races. This controversy indicates that racial differences in cognitive function are a complex issue that requires comprehensive consideration of multiple factors.

The underlying mechanism of the association between low muscle strength and increased cognitive function is not fully understood, but there may be multiple explanations. Firstly, muscle activity can improve human emotions and stimulate the brain to release substances beneficial to cognitive function (Battaglia et al. 2024, Gregorio and Battaglia 2024, Tanaka et al. 2024), such as endorphins and nerve growth factors. Low muscle strength may lead to reduced release of these substances, which in turn can affect cognitive function. Secondly, chronic low‐grade inflammation is one of the factors affecting the progression of sarcopenia. Chronic inflammation and oxidative stress are known triggers of neurodegenerative diseases, which not only damage muscle health but also accelerate cognitive decline (15, 17). In patients with sarcopenia, elevated levels of inflammatory factors such as TNF ‐α and IL–6 can be observed (Zabetian‐Targhi et al. 2021, Bahorik et al. 2024, Nazzi et al. 2024). These inflammatory factors not only affect muscle function but may also harm cognitive function by affecting the permeability of the blood‐brain barrier (14,19,23). Thirdly, hormones also play an important role in it. Older men generally have lower levels of testosterone in their bodies, and the decrease in testosterone is associated with decreased muscle strength and cognitive decline (Ali et al. 2018, Ebner et al. 2014). Insulin also plays an important role in regulating muscle function and cognitive function. Patients with sarcopenia have significantly lower levels of insulin in their bodies, and the deficiency of this hormone is associated with a net loss of protein and decreased muscle cell activity. Meanwhile, insulin can regulate synaptic transmission and plasticity in the central nervous system, which is crucial for memory and learning processes (Zhao et al. 2019, Ryan et al. 2021). Fourthly, the gut microbiota regulates cognitive behavior and brain function through the microbiota gut brain axis (Hu et al. 2016, Liu et al. 2021). Lipopolysaccharides and amyloid proteins secreted by gut bacteria can regulate signaling pathways, and disruption of gut microbiota may alter the permeability of the gut and blood‐brain barrier, thereby affecting cognitive function (Jiang et al. 2017). Finally, growth factors such as fibroblast growth factor (FGF) regulate muscle and bone quality during aging and also have neuroprotective effects (Jiang et al. 2017, Cho et al. 2022). Neurotrophic factors such as FGF‐23 (Hensel et al. 2016) can promote neuronal proliferation, maintain muscle homeostasis, and improve cognitive function.

Although this study provides important evidence of the association between grip strength and the risk of cognitive impairment, several limitations should be acknowledged. Firstly, the cross‐sectional design of NHANES data limits the causal inference between grip strength and cognitive impairment. Secondly, grip strength measurement may be affected by temporary health conditions, thereby affecting the stability of the measurement. Thirdly, although a series of confounding factors have been adjusted based on prior knowledge, there are still residual confounding factors from physical activity and dietary status that may affect the stability of the results. Finally, some covariates, such as alcohol consumption and smoking, rely on self‐reported data and introduce potential reporting biases.

## 6 Conclusion

This study reveals the association between low relative grip strength and increased risk of cognitive impairment in elderly Americans, highlighting that enhancing upper limb muscle strength may be an effective strategy for delaying cognitive decline. We deeply realize that this discovery not only enriches the understanding of the pathogenesis of cognitive impairment in theory but also provides a scientific basis for the health maintenance of the elderly in practical applications. However, in the specialized fields of cognitive function, such as orientation, memory, attention, and language, the specific effects still need to be further explored. Future research needs to expand sample diversity, cover multiple ethnic groups, and adopt prospective experimental designs to further consolidate this relationship. At the same time, the theoretical framework and research methods also need to be continuously optimized to comprehensively reveal the wide‐ranging impact and profound significance of this field.

## Author Contributions

**Wenyi Nie**: writing–original draft, methodology, visualization, investigation, data curation. **Jingqing Hu**: funding acquisition, writing–review and editing, project administration, resources, supervision.

## Ethics Statement

The protocol for NHANES received approval from the National Center for Health Statistics and its Ethics Review Board. All participants gave written informed consent. Since the data are publicly accessible, the need for an ethical approval statement and informed consent was exempted for this research.

### Peer Review

The peer review history for this article is available at https://publons.com/publon/10.1002/brb3.70381.

## Data Availability

The data that support the findings of this study are available from the corresponding author upon reasonable request.

## References

[brb370381-bib-0001] Ali, S. A. , T. Begum , and F. Reza . 2018. “Hormonal Influences on Cognitive Function.” Malaysian Journal of Medical Sciences 25, no. 4: 31–41. 10.21315/mjms2018.25.4.3. PMC6422548 30914845

[brb370381-bib-0002] Avila, J. F. , M. A. Renteria , R. N. Jones , et al. 2021. “Education Differentially Contributes to Cognitive Reserve Across Racial/Ethnic Groups.” Alzheimers Dement 17, no. 1: 70–80. 10.1002/alz.12176. 32827354 PMC8376080

[brb370381-bib-0003] Bahorik, A. L. , T. D. Hoang , D. R. Jacobs , D. A. Levine , and K. Yaffe . 2024. “Association of Changes in C‐Reactive Protein Level Trajectories through Early Adulthood with Cognitive Function at Midlife: The CARDIA Study.” Neurology 103, no. 2: e209526. 10.1212/WNL.0000000000209526. 38959452 PMC11226328

[brb370381-bib-0004] Battaglia, S. , A. Avenanti , L. Vecsei , and M. Tanaka . 2024. “Neurodegeneration in Cognitive Impairment and Mood Disorders for Experimental, Clinical and Translational Neuropsychiatry.” Biomedicines 12, no. 3: 574. 10.3390/biomedicines12030574. 38540187 PMC10968650

[brb370381-bib-0005] Bohannon, R. W 2019. “Grip Strength: An Indispensable Biomarker for Older Adults.” Clinical Interventions in Aging 14: 1681–1691. 10.2147/CIA.S194543. 31631989 PMC6778477

[brb370381-bib-0006] Boyle, P. A. , R. S. Wilson , N. T. Aggarwal , Y. Tang , and D. A. Bennett . 2006. “Mild Cognitive Impairment: Risk of Alzheimer Disease and Rate of Cognitive Decline.” Neurology 67, no. 3: 441–445. 10.1212/01.wnl.0000228244.10416.20. 16894105

[brb370381-bib-0007] Carson, J. L. , M. M. Brooks , P. C. Hebert , et al. 2023. “Restrictive or Liberal Transfusion Strategy in Myocardial Infarction and Anemia.” New England Journal of Medicine 389, no. 26: 2446–2456. 10.1056/NEJMoa2307983. 37952133 PMC10837004

[brb370381-bib-0008] Celis‐Morales, C. A. , P. Welsh , D. M. Lyall , et al. 2018. “Associations of Grip Strength With Cardiovascular, Respiratory, and Cancer Outcomes and All Cause Mortality: Prospective Cohort Study of Half a Million UK Biobank Participants.” British Medical Journal 361: k1651. 10.1136/bmj.k1651. 29739772 PMC5939721

[brb370381-bib-0009] Chai, S. , D. Zhao , T. Gao , et al. 2024. “The Relationship Between Handgrip Strength and Cognitive Function Among Older Adults in China: Functional Limitation Plays a Mediating Role.” Journal of Affective Disorders 347: 144–149. 10.1016/j.jad.2023.11.056. 37992778

[brb370381-bib-0010] Chamberlain, S. R. , J. Cavanagh , P. de Boer , et al. 2019. “Treatment‐Resistant Depression and Peripheral C‐Reactive Protein.” British Journal of Psychiatry 214, no. 1: 11–19. 10.1192/bjp.2018.66. PMC6124647 29764522

[brb370381-bib-0011] Chang, H. , and Y. Zhao . 2024. “Longitudinal Trajectories of Handgrip Strength and Their Association With Motoric Cognitive Risk Syndrome in Older Adults.” Arch Gerontol Geriat 120: 105334. 10.1016/j.archger.2024.105334. 38382231

[brb370381-bib-0012] Cho, S. , H. Lee , H. Y. Lee , S. J. Kim , and W. Song . 2022. “The Effect of Fibroblast Growth Factor Receptor Inhibition on Resistance Exercise Training‐Induced Adaptation of Bone and Muscle Quality in Mice.” Korean Journal of Physiology and Pharmacology 26, no. 3: 207–218. 10.4196/kjpp.2022.26.3.207. 35477548 PMC9046891

[brb370381-bib-0013] Dong, X. , S. Li , J. Sun , Y. Li , and D. Zhang . 2020. “Association of Coffee, Decaffeinated Coffee and Caffeine Intake From Coffee With Cognitive Performance in Older Adults: National Health and Nutrition Examination Survey (NHANES) 2011–2014.” Nutrients 12, no. 3: 840. 10.3390/nu12030840. 32245123 PMC7146118

[brb370381-bib-0014] Duchowny, K. A. , S. F. Ackley , W. D. Brenowitz , et al. 2022. “Associations Between Handgrip Strength and Dementia Risk, Cognition, and Neuroimaging Outcomes in the UK Biobank Cohort Study.” JAMA Network Open 5, no. 6: e2218314. 10.1001/jamanetworkopen.2022.18314. 35737388 PMC9227006

[brb370381-bib-0015] Ebner, N. C. , H. Kamin , V. Diaz , R. A. Cohen , and K. Macdonald . 2014. “Hormones as “Difference Makers” in Cognitive and Socioemotional Aging Processes.” Frontiers in Psychology 5: 1595. 10.3389/fpsyg.2014.01595. 25657633 PMC4302708

[brb370381-bib-0016] Filardi, M. , R. Barone , G. Bramato , et al. 2022. “The Relationship Between Muscle Strength and Cognitive Performance Across Alzheimer's Disease Clinical Continuum.” Frontiers in Neurology 13: 833087. 10.3389/fneur.2022.833087. 35645971 PMC9133788

[brb370381-bib-0017] Fotiadis, P. , L. Parkes , K. A. Davis , T. D. Satterthwaite , R. T. Shinohara , and D. S. Bassett . 2024. “Structure‐Function Coupling in Macroscale Human Brain Networks.” Nature Reviews Neuroscience 25, no. 10: 688–704. 10.1038/s41583-024-00846-6. 39103609

[brb370381-bib-0018] Frith, E. , and P. D. Loprinzi . 2018. “The Association Between Lower Extremity Muscular Strength and Cognitive Function in a National Sample of Older Adults.” Journal of Lifestyle Medicine 8, no. 2: 99–104. 10.15280/jlm.2018.8.2.99. 30474005 PMC6239135

[brb370381-bib-0019] Garcia, M. A. , B. Downer , C. T. Chiu , J. L. Saenz , K. Ortiz , and R. Wong . 2021. “Educational Benefits and Cognitive Health Life Expectancies: Racial/Ethnic, Nativity, and Gender Disparities.” The Gerontologist 61, no. 3: 330–340. 10.1093/geront/gnaa112. 32833008 PMC8023372

[brb370381-bib-0020] Gregorio, F. D. , and S. Battaglia . 2024. “The Intricate Brain‐Body Interaction in Psychiatric and Neurological Diseases.” Advances in Clinical and Experimental Medicine: Official Organ Wroclaw Medical University 33, no. 4: 321–326. 10.17219/acem/185689. 38515256

[brb370381-bib-0021] Hensel, N. , A. Schon , T. Konen , et al. 2016. “Fibroblast Growth Factor 23 Signaling in Hippocampal Cells: Impact on Neuronal Morphology and Synaptic Density.” Journal of Neurochemistry 137, no. 5: 756–769. 10.1111/jnc.13585. 26896818

[brb370381-bib-0022] Homer‐Bouthiette, C. , L. Xiao , and M. M. Hurley . 2021. “Gait Disturbances and Muscle Dysfunction in Fibroblast Growth Factor 2 Knockout Mice.” Scientific Reports‐UK 11, no. 1: 11005. 10.1038/s41598-021-90565-0. PMC8154953 34040128

[brb370381-bib-0023] Hu, X. , T. Wang , and F. Jin . 2016. “Alzheimer's Disease and Gut Microbiota.” Science China Life Sciences 59, no. 10: 1006–1023. 10.1007/s11427-016-5083-9. 27566465

[brb370381-bib-0024] Jiang, C. , G. Li , P. Huang , Z. Liu , and B. Zhao . 2017. “The Gut Microbiota and Alzheimer's Disease.” Journal of Alzheimer's Disease 58, no. 1: 1–15. 10.3233/JAD-161141. 28372330

[brb370381-bib-0025] Kim, G. R. , J. Sun , M. Han , C. M. Nam , and S. Park . 2019. “Evaluation of the Directional Relationship Between Handgrip Strength and Cognitive Function: The Korean Longitudinal Study of Ageing.” Age and Ageing 48, no. 3: 426–432. 10.1093/ageing/afz013. 30794286

[brb370381-bib-0026] Kim, J. , C. F. Mckenna , A. F. Salvador , et al. 2022. “Cathepsin B and Muscular Strength Are Independently Associated With Cognitive Control.” Brain Plasticity 8, no. 1: 19–33. 10.3233/BPL-210136. 36448041 PMC9661349

[brb370381-bib-0027] Kong, Y. , H. Xu , C. Li , Y. Yang , X. Zhu , and Y. Zuo . 2024. “Construction of PARI Public Health Education Programs for Chinese Undergraduates: A Delphi Study.” Front Public Health 12: 1390011. 10.3389/fpubh.2024.1390011. 38952723 PMC11215213

[brb370381-bib-0028] Li, T. , Z. Hu , L. Qiao , Y. Wu , and T. Ye . 2024. “Chronic Kidney Disease and Cognitive Performance: NHANES 2011–2014.” BMC Geriatrics 24, no. 1: 351. 10.1186/s12877-024-04917-2. 38637739 PMC11027402

[brb370381-bib-0029] Lim, G. , H. Lee , and Y. Lim . 2022. “Potential Effects of Resistant Exercise on Cognitive and Muscle Functions Mediated by Myokines in Sarcopenic Obese Mice.” Biomedicines 10, no. 10: 00. 10.3390/biomedicines10102529. PMC9599854 36289794

[brb370381-bib-0030] Liu, C. , W. H. Cheung , J. Li , et al. 2021. “Understanding the Gut Microbiota and Sarcopenia: A Systematic Review.” Journal of Cachexia, Sarcopenia and Muscle 12, no. 6: 1393–1407. 10.1002/jcsm.12784. 34523250 PMC8718038

[brb370381-bib-0031] Liu, C. , Q. Li , Z. Li , et al. 2024. “Association Between the Incident Hypertension Duration and Cognitive Performance in Older Adults: Data From the NHANES 2011–2014.” Aging Clinical and Experimental Research 36, no. 1: 181. 10.1007/s40520-024-02836-1. 39212760 PMC11364694

[brb370381-bib-0032] Liu, C. , K. Zhuang , D. C. Zeitlen , et al. 2024. “Neural, Genetic, and Cognitive Signatures of Creativity.” Communications Biology 2024;7, no. 1: 1324. 10.1038/s42003-024-07007-6. 39402209 PMC11473644

[brb370381-bib-0033] Lopez‐Bueno, R. , L. L. Andersen , J. Calatayud , et al. 2022. “Associations of Handgrip Strength With All‐Cause and Cancer Mortality in Older Adults: A Prospective Cohort Study in 28 Countries.” Age and Ageing 51, no. 5: afac117. 10.1093/ageing/afac117. 35639798 PMC9351371

[brb370381-bib-0034] Lopez‐Bueno, R. , J. Calatayud , L. L. Andersen , et al. 2023. “Dose‐Response Association of Handgrip Strength and Risk of Depression: A Longitudinal Study of 115 601 Older Adults From 24 Countries.” British Journal of Psychiatry 222, no. 3: 135–142. 10.1192/bjp.2022.178. PMC9929711 36464972

[brb370381-bib-0035] Ludyga, S. , U. Puhse , M. Gerber , and M. Mucke . 2021. “Muscle Strength and Executive Function in Children and Adolescents With Autism Spectrum Disorder.” Autism Research 14, no. 12: 2555–2563. 10.1002/aur.2587. 34351051 PMC9292567

[brb370381-bib-0036] Mao, Q. , X. Zhu , and Y. Kong . 2024. “Sleep Duration Mediates the Association Between Heavy Metals and the Prevalence of Depression: An Integrated Approach From the NHANES (2005‐2020).” Frontiers in Psychiatry 15: 1455896. 10.3389/fpsyt.2024.1455896. 39286395 PMC11404323

[brb370381-bib-0037] Mao, Q. , X. Zhu , X. Zhang , and Y. Kong . 2024. “Triglyceride‐Glucose Index and Its Combination With Obesity Indicators Mediating the Association Between 2‐Hydroxyfluorene and the Prevalence of Cardiovascular Disease: Evidence From the NHANES (2005‐2018).” Ecotoxicology and Environmental Safety 287: 117283. 10.1016/j.ecoenv.2024.117283. 39504874

[brb370381-bib-0038] Mavros, Y. , N. Gates , G. C. Wilson , et al. 2017. “Mediation of Cognitive Function Improvements by Strength Gains After Resistance Training in Older Adults With Mild Cognitive Impairment: Outcomes of the Study of Mental and Resistance Training.” Journal of the American Geriatrics Society 65, no. 3: 550–559. 10.1111/jgs.14542. 28304092

[brb370381-bib-0039] Nazzi, C. , A. Avenanti , and S. Battaglia . 2024. “The Involvement of Antioxidants in Cognitive Decline and Neurodegeneration: Mens Sana in Corpore Sano.” Antioxidants‐Basel 13, no. 6: 701. 10.3390/antiox13060701. 38929140 PMC11200558

[brb370381-bib-0040] Peng, T. C. , J. M. Chiou , Y. C. Chen , and J. H. Chen . 2024. “Handgrip Strength Asymmetry and Cognitive Impairment Risk: Insights From a Seven‐Year Prospective Cohort Study.” Journal of Nutrition, Health and Aging 28, no. 1: 100004. 10.1016/j.jnha.2023.100004.38267160

[brb370381-bib-0041] Peolsson, A. , R. Hedlund , and B. Oberg . 2001. “Intra‐and Inter‐Tester Reliability and Reference Values for Hand Strength.” Journal of Rehabilitation Medicine 33, no. 1: 36–41. 10.1080/165019701300006524. 11480468

[brb370381-bib-0042] Petersen, R. C 2016. “Mild Cognitive Impairment.” Continuum (Minneap Minn) 22, no. 2: 404–418. 10.1212/CON.0000000000000313. 27042901 PMC5390929

[brb370381-bib-0043] Ritchie, S. J. , E. M. Tucker‐Drob , J. M. Starr , and I. J. Deary . 2016. “Do Cognitive and Physical Functions Age in Concert From Age 70 to 76? Evidence From the Lothian Birth Cohort 1936.” The Spanish Journal of Psychology 19: E90. 10.1017/sjp.2016.85. 27917739

[brb370381-bib-0044] Ryan, A. S. , G. Li , S. Mcmillin , S. J. Prior , J. B. Blumenthal , and L. Mastella . 2021. “Pathways in Skeletal Muscle: Protein Signaling and Insulin Sensitivity After Exercise Training and Weight Loss Interventions in Middle‐Aged and Older Adults.” Cells‐Basel 10, no. 12: 3490. 10.3390/cells10123490. PMC8700073 34943997

[brb370381-bib-0045] Sanchez‐Rodriguez, D. , E. Marco , V. Davalos‐Yerovi , et al. 2019. “Translation and Validation of the Spanish Version of the SARC‐F Questionnaire to Assess Sarcopenia in Older People.” Journal of Nutrition, Health and Aging 23, no. 6: 518–524. 10.1007/s12603-019-1204-z.31233072

[brb370381-bib-0046] Su, Y. , C. Li , Y. Long , L. He , and N. Ding . 2022. “Association Between Sleep Duration on Workdays and Blood Pressure in Non‐Overweight/Obese Population in NHANES: A Public Database Research.” Scientific Reports‐UK 12, no. 1: 1133. 10.1038/s41598-022-05124-y. PMC8782988 35064191

[brb370381-bib-0047] Tan, V. , C. Chen , and R. A. Merchant . 2022. “Association of Social Determinants of Health With Frailty, Cognitive Impairment, and Self‐Rated Health Among Older Adults.” PLoS ONE 17, no. 11: e0277290. 10.1371/journal.pone.0277290. 36367863 PMC9651553

[brb370381-bib-0048] Tanaka, M. , S. Battaglia , L. Gimenez‐Llort , et al. 2024. “Innovation at the Intersection: Emerging Translational Research in Neurology and Psychiatry.” Cells‐Basel 13, no. 10: 790. 10.3390/cells13100790. PMC11120114 38786014

[brb370381-bib-0049] Van Hulle, C. , E. M. Jonaitis , T. J. Betthauser , et al. 2021. “An Examination of a Novel Multipanel of CSF Biomarkers in the Alzheimer's Disease Clinical and Pathological Continuum.” Alzheimers Dement 17, no. 3: 431–445. 10.1002/alz.12204. 33336877 PMC8016695

[brb370381-bib-0050] Wang, T. , R. Zheng , S. Zhang , et al. 2024. “Association Between Platelet‐to‐High‐Density Lipoprotein Cholesterol Ratio and Cognitive Function in Older Americans: Insights From a Cross‐Sectional Study.” Scientific Reports‐UK 14, no. 1: 25769. 10.1038/s41598-024-77813-9. PMC11519474 39468327

[brb370381-bib-0051] Wang, Z. , J. Wang , J. Guo , et al. 2023. “Association of Motor Function With Cognitive Trajectories and Structural Brain Differences: A Community‐Based Cohort Study.” Neurology 101, no. 17: e1718–e1728. 10.1212/WNL.0000000000207745. 37657942 PMC10624482

[brb370381-bib-0052] Xiao, Y. , J. J. Mann , J. C. Chow , et al. 2023. “Patterns of Social Determinants of Health and Child Mental Health, Cognition, and Physical Health.” Jama Pediatrics 177, no. 12: 1294–1305. 10.1001/jamapediatrics.2023.4218. 37843837 PMC10580157

[brb370381-bib-0053] Yeung, S. , E. M. Reijnierse , M. C. Trappenburg , et al. 2018. “Handgrip Strength Cannot Be Assumed a Proxy for Overall Muscle Strength.” Journal of the American Medical Directors Association 19, no. 8: 703–709. 10.1016/j.jamda.2018.04.019. 29935982

[brb370381-bib-0054] Zabetian‐Targhi, F. , V. K. Srikanth , K. J. Smith , et al. 2021. “Associations Between the Dietary Inflammatory Index, Brain Volume, Small Vessel Disease, and Global Cognitive Function.” Journal of the Academy of Nutrition and Dietics 121, no. 5: 915–924.e3. 10.1016/j.jand.2020.11.004. 33339764

[brb370381-bib-0055] Zhao, B. , T. Li , Y. Yang , et al. 2021. “Common Genetic Variation Influencing human White Matter Microstructure.” Science 372, no. 6548: eabf3736. 10.1126/science.abf3736. 34140357 PMC8370718

[brb370381-bib-0056] Zhao, F. , J. J. Siu , W. Huang , C. Askwith , and L. Cao . 2019. “Insulin Modulates Excitatory Synaptic Transmission and Synaptic Plasticity in the Mouse Hippocampus.” Neuroscience 411: 237–254. 10.1016/j.neuroscience.2019.05.033. 31146008 PMC6612444

[brb370381-bib-0057] Zhou, X. , J. Chen , Q. Zhang , et al. 2016. “Prognostic Value of Plasma Soluble Corin in Patients with Acute Myocardial Infarction.” Journal of the American College of Cardiology 67, no. 17: 2008–2014. 10.1016/j.jacc.2016.02.035. 27126527

[brb370381-bib-0058] Zou, Q. , X. Tian , Q. Mao , X. Zhu , and Y. Kong . 2024. “Lipid Accumulation Product Mediating the Association Between Uranium and Cerebrovascular Diseases Mortality: Evidence From National Health and Nutrition Examination Survey.” Medicine 103, no. 51: e40888. 10.1097/MD.0000000000040888. 39705492 PMC11666159

